# Monitoring Short-Term Moisture Progression in Superabsorbent Polymer-Modified Mortar Using Hygro-Electrical Measurements

**DOI:** 10.3390/polym18212609

**Published:** 2026-10-22

**Authors:** Borhan Uddin Rabbani, Jung Heum Yeon

**Affiliations:** Civil Engineering Program, Ingram School of Engineering, Texas State University, 601 University Drive, San Marcos, TX 78666, USA; borhan@txstate.edu

**Keywords:** superabsorbent polymers, moisture penetration, hygro-electrical measurements, reswelling, pore blocking

## Abstract

**Highlights:**

- 0.25 wt% SAP tended to resist moisture penetration compared to the control.
- SAP-modified mortar was~1.5 times more resistive than the control specimen.
- The electrical resistance front in SAP-modified mortar penetrated less than that in the control.
- Monitoring moisture movement in mortar by electrical resistance and internal relative humidity.

**Abstract:**

This study evaluates whether electrical resistance (ER) measurements can track short-term moisture-front progression in uncracked mortar modified with superabsorbent polymers (SAPs) under sustained hydrostatic pressure through comparison with internal relative humidity (IRH) measurements. The novelty lies in simultaneous, continuous monitoring at multiple depths using two independent sensing principles. Prism specimens (75 × 75 × 275 mm) containing control and 0.25 wt% acrylic acid-based SAP were monitored for 144 h under a hydrostatic pressure of approximately 0.01 MPa generated by a constant 1000-mm water head. One instrumented specimen was monitored for each mixture. At the 85% IRH threshold, the front reached 51 mm in the control and 43 mm in the SAP-modified mortar. The ER measurements were consistent with the moisture-front progression indicated by IRH measurements within each specimen. The measurements document specimen-specific IRH and electrical-resistance responses and motivate further investigation of depth-resolved moisture distributions in SAP-modified cement-based materials.

## 1. Introduction

The long-term durability of cement-based materials is strongly influenced by their resistance to moisture penetration because moisture serves as the primary transport medium for aggressive ions, such as chlorides, into the material matrix [1]. Moisture transport in cement-based materials is highly nonlinear and depends on factors such as moisture content, pore connectivity, and degree of saturation. Accordingly, penetration resistance can be evaluated directly by tracking the rate and depth of moisture front progression [1,2].

**Figure 1:**
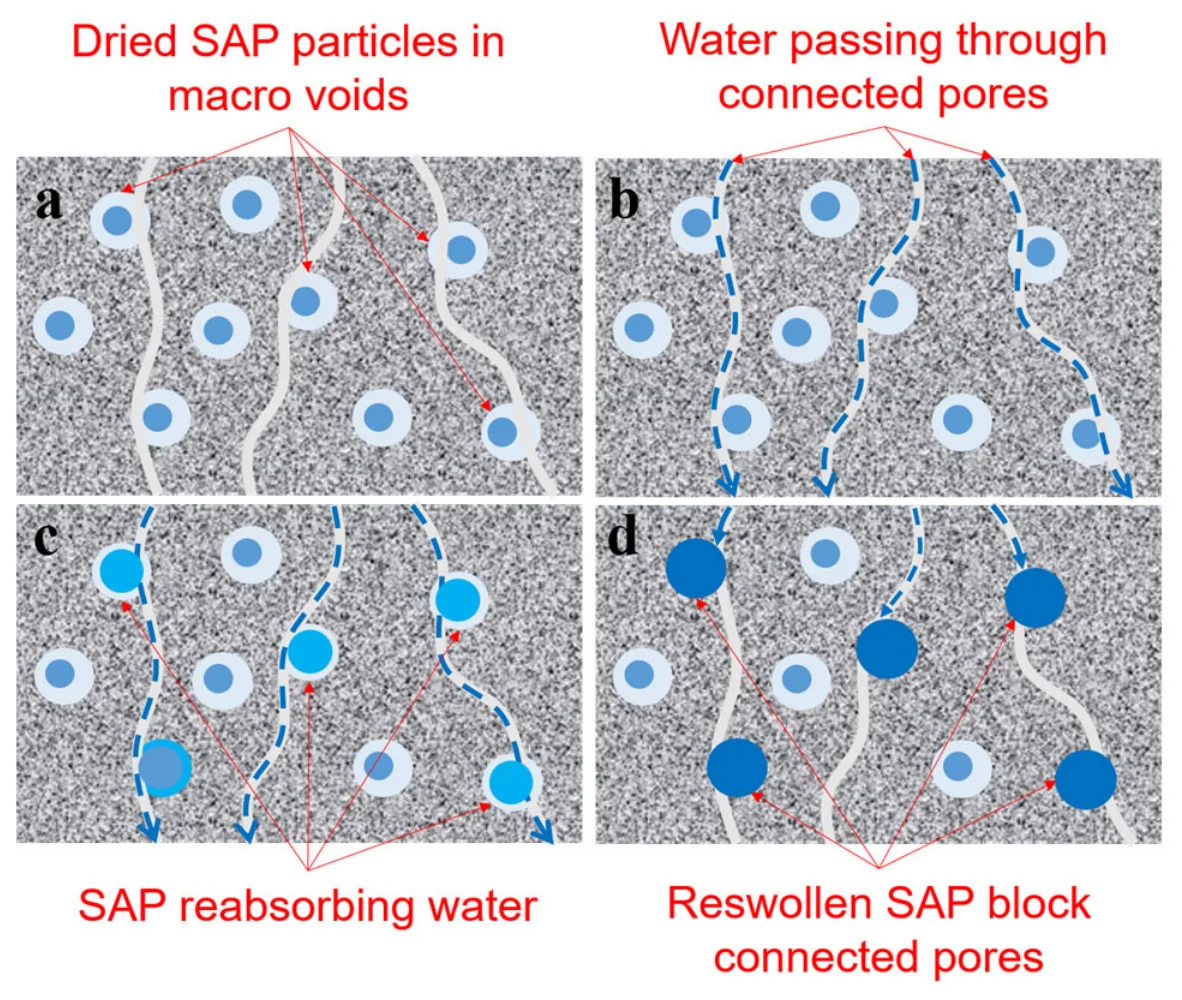
Conceptual illustration of SAP reswelling mechanism and resulting pore-blocking effect (not to scale).

Superabsorbent polymers (SAPs) are cross-linked hydrogels with considerable potential for the internal curing (IC) of cement-based materials [3,4]. During mixing, SAPs absorb pore solution and subsequently release the stored water as the internal relative humidity (IRH) decreases, thereby mitigating autogenous shrinkage and promoting continued hydration and densification of the surrounding cement paste [5,6]. SAPs can also influence mechanical performance. Although the voids remaining after water release may reduce compressive strength, this adverse effect can be partially offset by the enhanced hydration provided through internal curing [7]. Through continued hydration, pore refinement, and repeated reswelling upon water exposure, SAPs may also reduce water penetration, depending on their dosage and curing conditions, as illustrated in Figure 1 [8]. Figure 1 is a conceptual schematic illustrating the potential mechanisms underlying the pore-blocking effect of SAPs [9,10].

Water penetration in SAP-modified mortar has previously been investigated using neutron radiography [11]. Although this technique provides high-resolution spatial maps of moisture distribution, it requires access to specialized, large-scale facilities and is generally limited to relatively small specimens. Moreover, it is not readily adaptable to continuous, in-situ monitoring at multiple depths under sustained water exposure. Such spatial and temporal tracking of an advancing moisture front requires non-destructive measurements collected continuously at several depths. IRH sensors primarily reflect vapor-phase humidity [12], whereas electrical resistance (ER) measurements respond to the volume, conductivity, and connectivity of liquid phasewithin the pore network [13]. The two methods track the advancing moisture front through complementary mechanisms, i.e., vapor-phase humidity and liquid-phase electrical conduction, providing complementary measures of moisture progression within the same specimen. Depth-resolved electrical measurements are an established approach for monitoring moisture ingress in cementitious materials [14]. McCarter et al. [15] monitored transient moisture profiles in cover-zone concrete using conductivity measurements at discrete depths and derived capillary transport coefficients from the resulting profiles. Rajabipour et al. [16] developed a procedure relating conductivity measured between embedded electrodes to the position of the moisture front during rewetting. The configuration used in this study applies this established measurement principle to SAP-modified mortar, along with simultaneous IRH monitoring at multiple depths.

To address these limitations, this study uses embedded IRH sensors and electrodes to continuously track moisture-front progression in control and 0.25 wt% acrylic acid-based SAP-modified mortars subjected to a controlled wetting protocol. Standard gravimetric capillary absorption testing measures cumulative water uptake through periodic weighing, providing a bulk measure rather than a depth-resolved moisture profile. In contrast, this study continuously records IRH and ER at seven fixed depths without removing the specimens, allowing the progression of selected measurement thresholds to be followed over time. Furthermore, the sustained 1000 mm water head corresponds to approximately 0.01 MPa of pressure at the exposed surface, introducing a pressure boundary condition in addition to capillary suction rather than modifying the transport regime [17]. The sustained hydrostatic head represents a simplified exposure condition relevant to prolonged contact with standing water or shallow groundwater.

The objective of this study is to evaluate whether embedded IRH and ER sensors can resolve short-term moisture-front progression at multiple depths and to document their responses in a control specimen and an SAP-modified specimen subjected to the same wetting protocol. The novelty of this study lies in the simultaneous, continuous monitoring of moisture-front progression at multiple depths using two independent sensing principles. By investigating moisture ingress, this study provides insights into the durability of SAP-modified cement-based materials, whose long-term performance is governed not only by bulk mechanical strength but also by environmental exposure and the ingress of moisture [1].

## 2. Materials and Methods

### 2.1. Materials

Two mortar mixtures were prepared using Type I ordinary Portland cement (specific gravity: 3.15), locally sourced sand (specific gravity: 2.60; fineness modulus: 2.87), and tap water at an effective water-to-cement ratio (w/c) of 0.485. One control specimen and one SAP-modified specimen, each measuring 75 × 75 × 275 mm, were cast. The SAP-modified mortar contained 0.25 wt% acrylic acid-based SAP by cement mass (BASF, Florham Park, NJ, USA; mean particle size: approximately 470 μm). One instrumented specimen per mixture was continuously monitored at multiple depths, with the experimental program focusing on the evolution of the measured responses within each specimen. The mixture proportions are presented in Table 1.

The absorption capacity of SAP measured in deionized water, tap water, and a 0.9% NaCl solution (to simulate absorption in the presence of chloride ions) using the teabag test [18] was 176.8 g/g, 130 g/g, and 52.8 g/g, respectively. Under mixing conditions, the equivalent-flow method, in which the flow of the SAP-modified mortar was matched to that of the control [7], indicated an absorption capacity of 9.25 g/g because of the high ionic strength of the pore solution [19,20]. Additional IC water was therefore added based on this measured absorption capacityto achieve the target effective w/c.

### 2.2. Instrumentation

**Figure 2:**
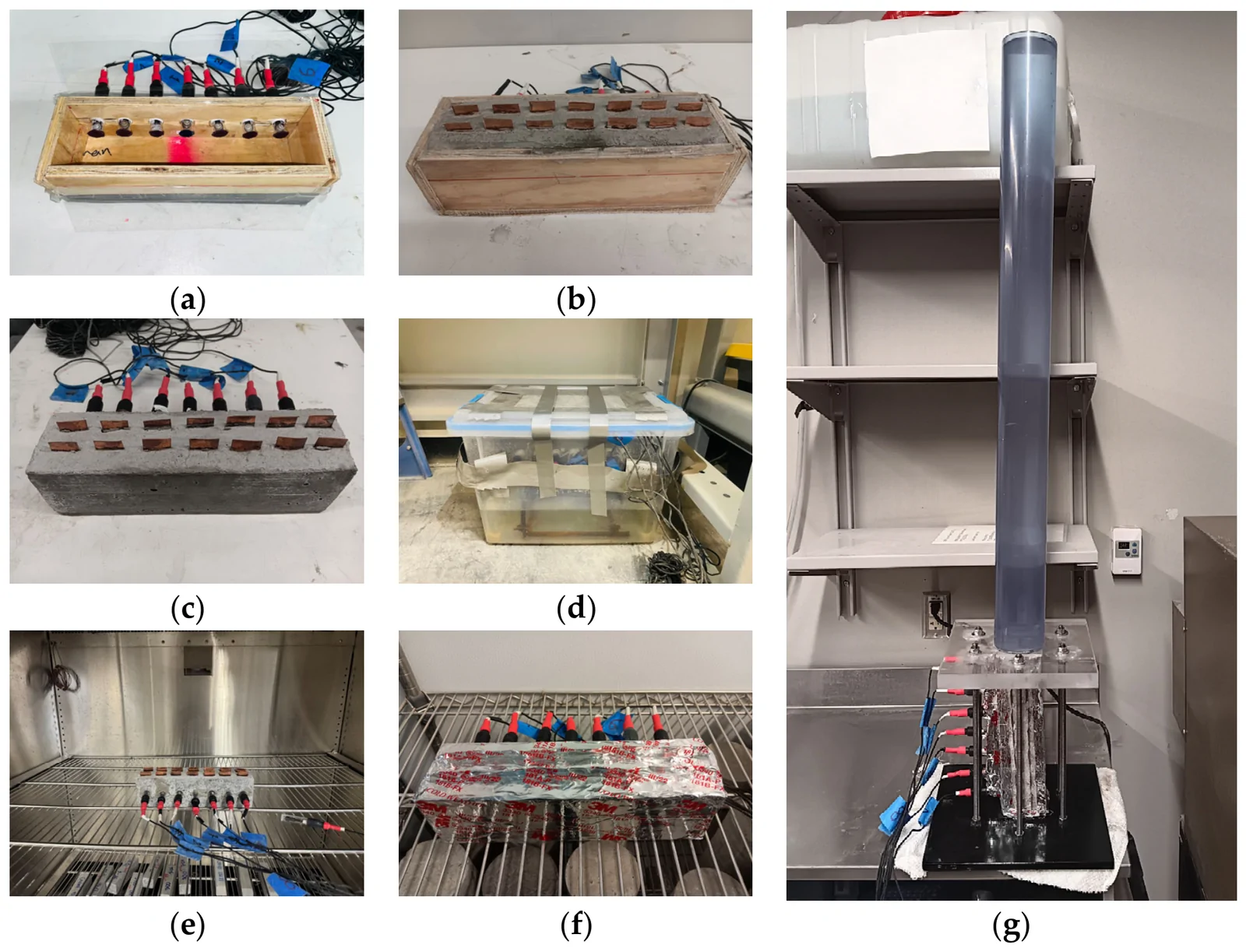
Experimental setup: (**a**) wooden mold with embedded IRH sensors; (**b**) specimen after 24 h of curing; (**c**) specimen after demolding; (**d**) specimen stored in a high-humidity plastic chamber during 21-day moist curing; (**e**) specimen in the environmental chamber during drying; (**f**) specimen in the environmental chamber during stabilization; and (**g**) wetting-test setup.

Seven IRH sensors (15 mm in diameter) were positioned at the specimen mid-height (37.5 mm) and embedded 15 mm into the specimen. Seven pairs of copper electrodes (25 × 75 × 0.81 mm) were installed along the longitudinal axis at a center-to-center spacing of 37.5 mm, with the center of the first sensor located 25 mm from the wetting face. The electrodes were embedded to a depth of 65 mm from the top surface, with a 25 mm spacing between the two electrodes in each pair (Figure 2a,b). IRH was recorded using a multichannel data logger (MSHTDL-16, Ymatic, Saitama, Japan; ±2% accuracy). ER was measured using an inductance-capacitance-resistance (LCR) meter (LCR-6200, GW Instek, New Taipei City, Taiwan) at a frequency of 1 kHz and an excitation voltage of 1.00 V. A four-probe configuration was used to eliminate contact-resistance effects, while the applied alternating current minimized electrode polarization [21]. Copper electrodes were selected because of their high electrical conductivity, ready availability, and use in previous studies monitoring cement-based materials [22]. The test was conducted under isothermal conditions at 23 °C. Two-dimensional ER contour plots were generated in OriginPro using measurement time, sensor depth, and measured ER as the X, Y, and Z variables, respectively. Interpolation between the discrete measurements was used to visualize ER variations with depth and time. Each specimen’s contour plot was scaled to its own recorded resistance range, as absolute resistance values are not directly comparable between the two mixtures.

### 2.3. Conditioning and Wetting Protocol

The specimens were subjected to a drying-stabilization-wetting sequence. After demolding at 24 h, they were moist-cured for 21 days in a high-humidity plastic chamber maintained at approximately 100% RH, as monitored using IRH sensors, until near-saturated conditions were achieved. The specimens were then dried at 35 °C and approximately 25% RH until both mixtures reached a comparable IRH of approximately 65–70%, thereby ensuring similar initial moisture conditions before wetting [23]. Comparable IRH values do not, however, establish comparable moisture contents or degrees of saturation, as further discussed in Section 3 and Section 4. A previous study reported that drying-induced microcracking is strongly temperature-dependent and is largely confined to approximately 10 mm from the exposed surface when cementitious materials are dried at temperatures ranging from 50 °C to 105 °C [24]. The lower drying temperature of 35 °C used in the present study was therefore expected to limit drying-induced damage. Both mixtures were subjected to identical drying conditions.

Following drying, the lateral surfaces of the specimens were sealed, and the specimens were stored at 23 °C and 50% RH to allow internal moisture redistribution until quasi-stable IRH profiles were achieved. The four longitudinal faces and the face opposite the wetting surface were sealed with adhesive aluminum tape, leaving only one 75 × 75 mm end face exposed to water. This arrangement was intended to promote one-dimensional moisture transport along the specimen length. The control and SAP-modified specimens required stabilization periods of 6 and 8 days, respectively. In the wetting stage, a single 75 × 75 mm end face was exposed to a constant water head of 1000 mm, resulting in a ~0.01 MPa hydrostatic pressure at 23 °C. IRH and ER were continuously recorded for 144 h (Figure 2c–g).

## 3. Results and Discussion

### 3.1. IRH Front Behavior

**Figure 3:**
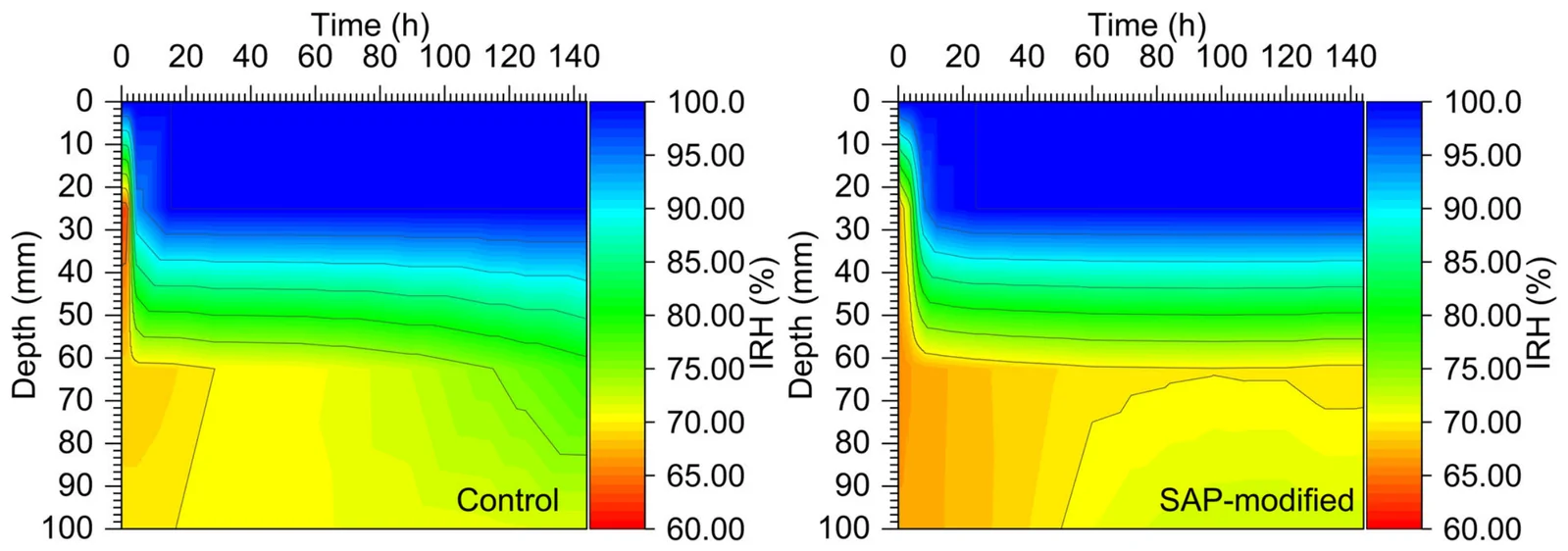
IRH changes in control and SAP-modified mortar.

The IRH contours in Figure 3 revealed distinct moisture-penetration behavior between the two mixtures. In the near-surface region, the near-saturated zone, defined as IRH ≥ 95%, extended to depths of 33 mm in the control mortar and 31 mm in the SAP-modified mortar. The moisture-front depth was defined as the location at which the IRH first exceeded a specified threshold, based on linear interpolation of the depth-time contours. At the 85% IRH threshold, the advancing front reached 51 mm in the control mortar but only 43 mm in the SAP-modified mortar. At each measurement time, moisture-front depth was determined from the IRH depth profile used to generate the contours. It was defined as the shallowest depth at which the profile crossed below the specified IRH threshold (85% or 95%). This depth was calculated by linear interpolation between the two adjacent sensors whose readings bracketed the threshold. The 95% IRH threshold was selected to indicate near-saturated conditions while accounting for reduced sensor reliability near saturation [25]. The 85% IRH threshold was selected empirically as a reference for comparing moisture progression in the partially saturated zone, as this level reached approximately the depth of Sensor 2 (~50 mm) during the monitoring period. Thus, the difference between the mixtures increased from 2 mm at the 95% threshold to 8 mm at the 85% threshold, indicating that the effect of SAP incorporation became more pronounced with increasing penetration depth.

The SAP-modified mortar, therefore, maintained a markedly drier interior and exhibited greater resistance to moisture penetration, even at a relatively low SAP dosage of 0.25 wt%. This behavior is consistent with SAP-induced pore refinement and reswelling [6]. The contour data indicate delayed moisture-front progression in the SAP-modified mortar under the tested conditions. This observation alone does not establish a lower moisture diffusivity or an intrinsic reduction in penetrability. Differences in initial moisture conditions may also have contributed to the observed behavior. Additional controlled transport measurements are needed to quantify these properties and evaluate the proposed pore-blocking mechanism. The present study demonstrates the same general trend under sustained hydrostatic pressure of approximately 0.01 MPa in uncracked mortar, using continuous embedded monitoring at multiple depths, rather than short-term capillary absorption testing of cracked, oven-dried specimens. Although the SAP-modified mortar required a somewhat longer drying period than the control mortar to reach the target IRH range of approximately 65–70%, both mixtures were subjected to identical drying conditions and entered the wetting stage with comparable internal moisture levels; the longer conditioning period reflects the greater moisture-retention capacity and gradual moisture release associated with SAP incorporation. The longer conditioning period of the SAP-modified mortar may have contributed to further hydration and matrix development, as well as some variation in its initial moisture condition. Comparable initial IRH values may not fully reflect differences in moisture content or degree of saturation. Conditioning history may therefore have influenced the observed moisture-front progression alongside SAP incorporation.

### 3.2. ER Front Behavior

**Figure 4:**
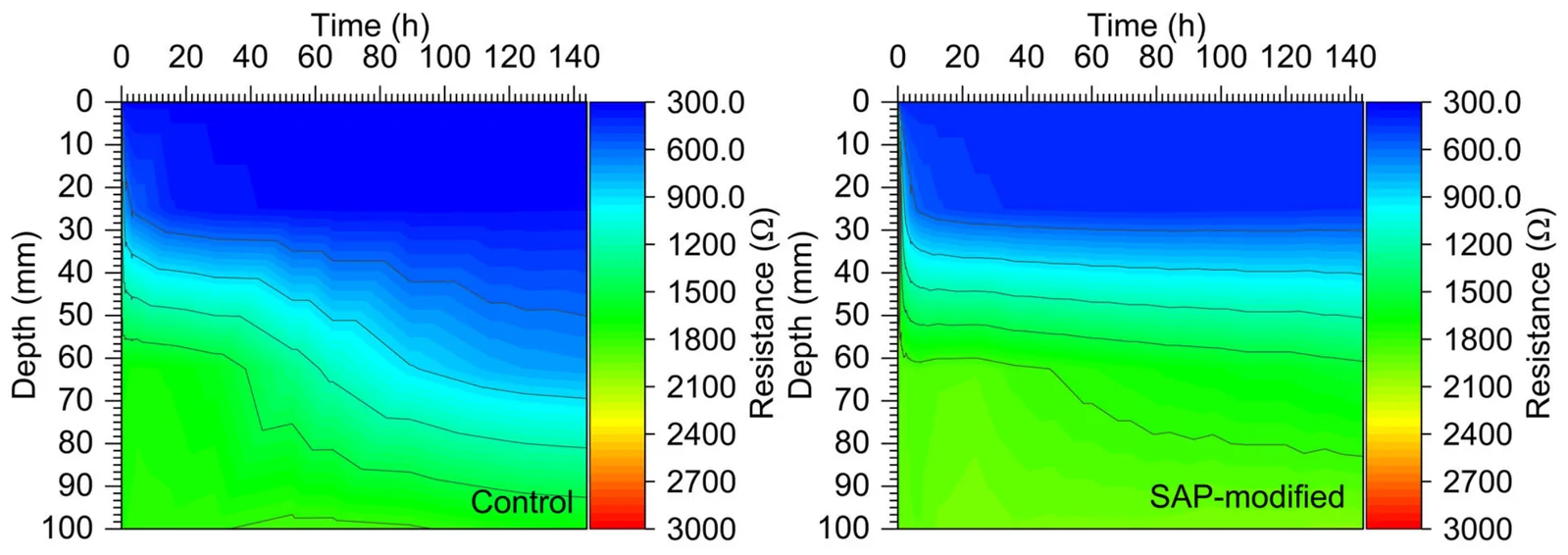
Electrical resistance changes in control and SAP-modified mortar.

The ER contours in Figure 4 track the advancing moisture front within each specimen through a complementary, conduction-based measurement. Each panel is scaled to the resistance range recorded in that specimen and referenced to the minimum ER measured at its wetted face. Because absolute resistance depends on pore-solution chemistry, pore structure, conditioning history, and moisture state, differences in resistance magnitude between the two specimens are treated as descriptive observations rather than evidence of a validated mixture-dependent trend. Both specimens exhibited the same general response: resistance decreased first at the shallowest electrode pair, and the low-resistance region extended progressively inward during the 144 h wetting period. In the control specimen, this region continued to expand throughout the test, whereas the SAP-modified specimen exhibited limited further advancement after the initial moisture uptake. At 144 h, both specimens showed a relatively sharp resistance contrast between the wetted and unwetted regions. These observations suggest slower subsequent moisture-front progression in the SAP-modified specimen under the tested conditions.

The selected ER thresholds provide common reference levels for comparing the electrical responses of the two specimens. However, mixture-specific relationships between ER and moisture condition were not established, so the same resistance may correspond to different moisture conditions in the two mixtures [13]. Accordingly, the reported 42.1% reduction in the depth of the 900 Ω contour reflects a difference in electrical response and may not directly correspond to an equivalent reduction in moisture penetration. ER monitoring began at the onset of wetting, without a separate pre-wetting baseline. ER contours may be influenced by moisture conditions, pore structure, pore-solution chemistry, and temperature. Differences in ER contour depth between the mixtures should therefore be interpreted cautiously, as their relationship to moisture-front depth or moisture content has not been independently calibrated.

After approximately 60 h, the selected IRH and ER contours appeared to advance more rapidly. One possible explanation is increased connectivity of water-filled pores as wetting progressed. For ER, a nonlinear response to increasing saturation could also produce a relatively abrupt resistance decrease, causing the selected contour levels to indicate apparent acceleration [26,27]. This electrical response alone, however, does not explain the corresponding IRH behavior. The inferred progression may also be influenced by the discrete sensor spacing and interpolation between measurement locations. Hydrostatic-pressure-induced microcracking is considered less likely given the relatively low applied pressure (~0.01 MPa), although cracking was not independently assessed. These explanations remain hypotheses requiring further investigation.

At 144 h, the separation between the 900 Ω fronts was 29 mm, whereas the difference between the corresponding ≥85% IRH fronts was approximately 8 mm. This ER response is consistent with published observations from depth-resolved electrical monitoring of water ingress in control specimens. McCarter et al. [15] measured conductivity using seven electrode pairs positioned between 10 and 175 mm from the exposed concrete surface, employing a two-point method with a 365 mV signal at 1 kHz. They reported a well-defined change in conductivity at each electrode pair as the moisture front entered the region between the electrodes. Guo et al. [28] investigated water permeability in concrete incorporating SAP at dosages of 0.2–0.6% and reported a 35–45% reduction in water penetration, which they attributed to a pore-blocking effect. Accordingly, the limited moisture-front advancement observed in the SAP-modified specimen in the present study may reflect a similar mechanism. However, this interpretation remains a hypothesis requiring further investigation, particularly given the differences in conditioning duration between the studies. The ER differences may reflect the distinct physical quantities measured by the two methods. Bulk ER depends on pore solution conductivity, pore structure and connectivity, and degree of saturation [29]. At comparable saturation and pore structure, a more conductive pore solution would produce lower resistance [30]. Conversely, differences in pore solution chemistry could produce different ER values even at similar moisture contents. Because SAP may influence each of these factors, the ER differences between the mixtures should be interpreted as a combined response. Independent pore solution and pore structure measurements would help distinguish their respective contributions.

## 4. Conclusions and Recommendations

This study suggests that incorporating 0.25 wt% SAP may reduce moisture penetration in mortar subjected to a hydrostatic pressure of approximately 0.01 MPa. Both the IRH and ER measurements indicated slower moisture-front progression in the SAP-modified specimen under the conditioning and exposure conditions examined. The SAP-modified specimen also maintained lower interior IRH values than the control specimen, although this does not necessarily indicate a lower moisture content or degree of saturation.

Despite these findings, this study has the following limitations:

• The findings reflect the responses of one instrumented specimen per mixture, and specimen-to-specimen variability remains to be assessed. Replicate testing would help establish the reproducibility of the observed trends and provide a stronger basis for quantifying the influence of SAP incorporation.
• Front depths were estimated by linear interpolation between sensors spaced 37.5 mm apart and may be influenced by sensor measurement uncertainty and the assumed linear variation between measurement locations. Although uncertainty in the estimated depths was not quantified and their positions were not independently validated, these estimates provide a basis for comparison, with small differences, such as approximately 2 mm, warranting cautious interpretation.
• Differences in initial moisture conditions may have influenced moisture-front progression and should therefore be considered when interpreting the differences observed between the mixtures [31].
• The difference in conditioning/drying duration between the mixtures may have influenced ER independently of subsequent moisture penetration and should be considered when interpreting the comparison between the mixtures.
• Ionic interactions involving the acrylic-based SAP may influence pore solution conductivity and, consequently, ER independently of moisture content [32].
• The additional internal curing water may influence microstructural development and matrix porosity [33]. Further porosity characterization would help clarify its contribution to the observed moisture-transport behavior.
• The relative contributions of continued matrix hydration and physical pore blocking by SAP remain to be clarified through targeted microstructural and transport measurements.

Taken together, these limitations mean that the present measurements do not isolate the effect of SAP on water ingress. The reported differences should therefore be interpreted as the responses of two individually monitored specimens, rather than as an established effect of SAP incorporation. Future work should include independently prepared replicate specimens for each mixture to quantify specimen-to-specimen variability and assess reproducibility. Uncertainty in the estimated IRH-threshold positions should be evaluated by considering sensor accuracy, sensor spacing, and interpolation assumptions. These estimates should also be evaluated against independently measured spatial moisture profiles, using techniques such as time-resolved two-dimensional ^1^H NMR imaging [34]. Both mixtures should undergo a consistent curing and conditioning protocol, with their initial moisture contents or degrees of saturation determined independently rather than inferred from IRH alone. Establishing mixture-specific relationships between electrical resistance and degree of saturation would further support the interpretation of the electrical-resistance contours in terms of moisture condition.

## Figures and Tables

**Table 1:** Mixture proportions used in this study (unit: kg/m^3^).

Materials	Cement	Sand	Water	SAP	IC Water
Control	527.1	1449.5	255.6	0	0
SAP-modified	527.1	1449.5	255.6	1.32	12.3

## Data Availability

Data will be made available on request.

## Abbreviations

The following abbreviations are used in this manuscript:

SAP	Superabsorbent polymer
IRH	Internal relative humidity
ER	Electrical resistance
RH	Relative humidity
IC	Internal curing
